# Combined Supplementation of Coenzyme Q_10_ and Other Nutrients in Specific Medical Conditions

**DOI:** 10.3390/nu14204383

**Published:** 2022-10-19

**Authors:** Torsak Tippairote, Geir Bjørklund, Amin Gasmi, Yuliya Semenova, Massimiliano Peana, Salvatore Chirumbolo, Tony Hangan

**Affiliations:** 1 Department of Nutritional and Environmental Medicine, HP Medical Center, Bangkok 10540, Thailand; 2 Council for Nutritional and Environmental Medicine, Toften 24, 8610 Mo i Rana, Norway; 3 Société Francophone de Nutrithérapie et de Nutrigénétique Appliquée, 69100 Villeurbanne, France; 4 School of Medicine, Nazarbayev University, Astana 020000, Kazakhstan; 5 Department of Chemical, Physical, Mathematical and Natural Sciences, University of Sassari, via Vienna 2, 07100 Sassari, Italy; 6 Department of Neurosciences, Biomedicine and Movement Sciences, University of Verona, 37134 Verona, Italy; 7 CONEM Scientific Secretary, Strada Le Grazie 9, 37134 Verona, Italy; 8 Faculty of Medicine, Ovidius University of Constanta, 900470 Constanta, Romania

**Keywords:** coenzyme Q_10_, dietary supplements, ubiquinone, mitochondria, bioenergetics, combined supplements

## Abstract

Coenzyme Q_10_ (CoQ_10_) is a compound with a crucial role in mitochondrial bioenergetics and membrane antioxidant protection. Despite the ubiquitous endogenous biosynthesis, specific medical conditions are associated with low circulating CoQ_10_ levels. However, previous studies of oral CoQ_10_ supplementation yielded inconsistent outcomes. In this article, we reviewed previous CoQ_10_ trials, either single or in combination with other nutrients, and stratified the study participants according to their metabolic statuses and medical conditions. The CoQ_10_ supplementation trials in elders reported many favorable outcomes. However, the single intervention was less promising when the host metabolic statuses were worsening with the likelihood of multiple nutrient insufficiencies, as in patients with an established diagnosis of metabolic or immune-related disorders. On the contrary, the mixed CoQ_10_ supplementation with other interacting nutrients created more promising impacts in hosts with compromised nutrient reserves. Furthermore, the results of either single or combined intervention will be less promising in far-advanced conditions with established damage, such as neurodegenerative disorders or cancers. With the limited high-level evidence studies on each host metabolic category, we could only conclude that the considerations of whether to take supplementation varied by the individuals’ metabolic status and their nutrient reserves. Further studies are warranted.

## 1. Introduction

In 1957, Crane et al. isolated a new quinone compound, initially referred to as Q-275, from beef heart mitochondria and described its ability to undergo reversible oxidation and reduction [1]. With its functional quinone moiety and its ubiquitous presence in living cells, this compound was later officially named ‘ubiquinone’ by the IUPAC-IUB Commission on Biochemical Nomenclature [2,3]. However, there were other common names for ubiquinone, including Coenzyme Q_10_ (CoQ_10_), CoQ, ubiquinone-Q10, vitamin Q10, and ubidecarenone [4].

CoQ_10_ consists of two functional groups, i.e., the five-carbon isoprene ‘tail’ and the benzoquinone ‘head’. The lipid-soluble tail consists of ten isoprenes, with a total of 50 carbon atoms, hence the name ‘CoQ_10_’ [5,6]. Humans can internally synthesize CoQ_10_. The synthesis of the benzoquinone head requires amino acids, either tyrosine or phenylalanine, while the mevalonate pathway provides the acetyl coenzyme A for the polyisoprenoid tail synthesis [5,7,8]. The CoQ_10_ head and tail are synthesized in the cytosol but the joining of both functional groups occurs in the mitochondria [5,9]. The CoQ_10_ biosynthesis requires support from various macro- and micronutrients, such as pantothenic acid for the CoA precursor, pyridoxine for the benzoquinone production, and s-adenosyl methionine for methylation support and isoprene production [10].

Humans can internally synthesize CoQ_10_, while the dietary CoQ_10_ sources additionally contribute to its total tissue pool. The main dietary sources of CoQ_10_ include fish and meat, while vegetables generally contain low CoQ_10_ quantity. Dietary CoQ_10_ sparsely absorbs in the hydrophilic intestinal environment due to its lipophilic and high molecular weight properties. Following the gut uptake, CoQ_10_ circulates in the lymphatic system and ultimately drains into the blood circulation [11]. Consequently, the CoQ_10_ level in the liver and plasma lipoproteins appears shortly after absorption, but the elevation of plasma CoQ_10_ level varies by the blood cholesterol and lipoprotein concentrations [6,12]. The CoQ_10_ turnover in the body is relatively fast, with a 49 to 125 h half-life, depending on the tissue type [13].

The combination of endogenous biosynthesis and dietary intake is largely sufficient to prevent the CoQ_10_ deficiency state in a healthy individual [14,15]. However, cumulative studies reported the association of low CoQ_10_ levels in specific conditions such as following strenuous exercise, during aging, after taking some prescribed medications, in patients with various metabolic disorders, and in individuals with cancers [6,8,16,17,18,19,20,21,22]. Despite the documented low CoQ_10_ levels, the clinical outcomes of CoQ_10_ interventions, either single or in combination with other nutrients, were generally inconsistent. In this review, we explored the previous CoQ_10_ clinical trials, both single and combined supplementations, in specific medical conditions and deduced whether the differences in host metabolic status influence the CoQ_10_ interventional outcomes.

## 2. Materials and Methods

### 2.1. Search Strategy

From the available public databases up to the date 10 January 2021, we initially acquired 256 publications by using the Medical Subject Heading (MeSH) Ubiquinone, Dietary Supplements, and randomized controlled trials, together with the truncated keywords Coenzyme Q* and supplement*. In the PubMed database, we used the following search query: (((“Ubiquinone”(MeSH)) OR (Coenzyme Q*(tw))) AND ((“Dietary Supplements” (MeSH)) OR (supplement*(tw)))) AND (“randomized controlled trials as topic”(MeSH)). We also retrieved additional articles from Cochrane Library, Scopus, Google Scholar, ResearchGate, and relevant citation searches.

### 2.2. Study Screening, Selection, and Inclusion

We initially identified the studies by their titles and abstracts for their compliance with the following inclusion criteria: (i) controlled clinical trials on the effects of CoQ_10_ and its analogs with or without the addition of other nutrients; (ii) controlled clinical trials that addressed the effects of CoQ_10_ and its analogs in a range of medical conditions; (iii) studies for which the full texts were available to enable a comprehensive review.

With all retrieved full texts of articles, we screened and grouped them according to participants’ status or medical conditions. We then evaluated their suitability for inclusion in the present review. We also acquired additional studies from the relevant citation searches.

**Figure 1:**
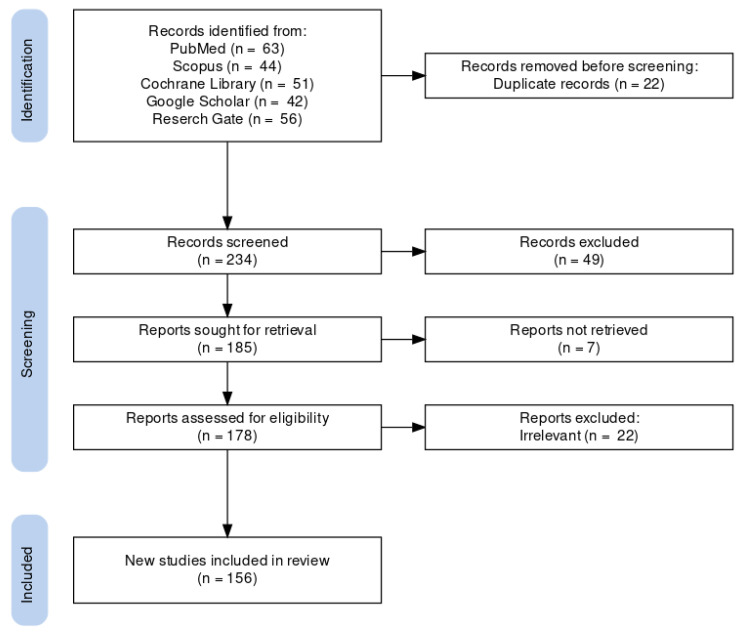
The PRISMA flow diagram.

After excluding the duplicated, irrelevant, and no full-text-available articles, we included 156 studies in this narrative review. We generated a PRISMA diagram to describe the flow of information through the processes of identification, screening, and including records in this literature review, as shown in Figure 1 [23,24].

## 3. Physiological Roles of CoQ_10_ in Humans

### 3.1. CoQ_10_ Roles in Mitochondrial Bioenergetics

CoQ_10_ roles are crucial to the mitochondrial respiratory chain as the electron acceptor. It modulates the electron transferring from the bioenergetic-derived reducing equivalents, i.e., nicotinamide adenine dinucleotide (NADH) and flavin adenine dinucleotide (FADH_2_), through the complex I, II, and III in the electron transport chain (ETC). The concurrent proton flow, from the mitochondrial matrix to the intermembrane space, generates the intermembrane proton gradients, which are essential for oxidative phosphorylation and subsequent adenosine triphosphate (ATP) synthesis [5]. Accordingly, CoQ_10_ intervention could have potential bioenergetic benefits in clinical conditions with mitochondrial dysfunction.

While mitochondria are the bioenergetic hub of the cells, they are also the predominant source of reactive oxygen species (ROS) production, oxidative stress, and immunologic and apoptotic regulation. The imbalances of these physiological processes underly diverse metabolic conditions [25]. As the critical supporter of mitochondrial functions, the importance of CoQ_10_ might extend beyond bioenergetics.

### 3.2. CoQ_10_ Role as an Antioxidant

With its reversible redox potential and membrane-associated locations, CoQ_10_ renders antioxidant protection to the organelles and cell lipid membranes [13,26,27,28]. Inside the cells, the benzoquinone head of CoQ_10_ exists in three interchangeable oxidation states, i.e., the fully reduced ubiquinol (CoQ_10_H_2_), the ubisemiquinone intermediate (CoQ_10_H•), and the fully oxidized ubiquinone (CoQ_10_). These redox states are culpable for the scavenging of ROS as well as the mediation of electrons transferring in the mitochondrial ETC.

**Figure 2:**
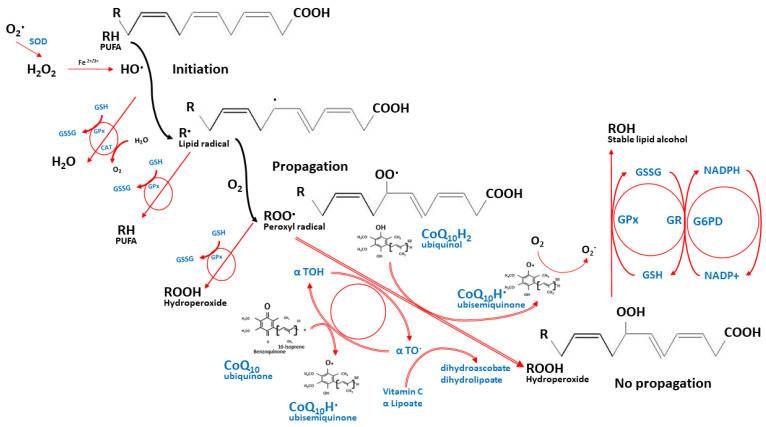
The integrated antioxidant defenses against lipid peroxidation. The nutrient network halts the propagation of lipid peroxyl radicals while their redox interactions maintain their reduction states. These nutrients include coenzyme Q_10_, α tocopherol, vitamin C, α lipoic acid, glutathione, and the micronutrients that support the activities of antioxidant enzymes such as selenium, manganese, copper, and zinc (not shown in the figure). O_2_^•^—superoxide anion radical, H_2_O_2_—hydrogen peroxide, Fe^2+/3+^—ferrous or ferric iron, PUFA—polyunsaturated fatty acids, GSH—reduced glutathione, GSSG—glutathione disulfide, GPx—glutathione peroxidase, CAT—catalase, H_2_O—water, O_2_—oxygen, α TOH—reduced α tocopherol, α TO^•^—oxidized α tocopherol.

Nevertheless, the integrated redox modulation of CoQ_10_ requires support from other nutrients, specifically α-tocopherol, vitamin C, and other micronutrients. Figure 2 depicts this integrated antioxidant network of CoQ_10_ against lipid peroxidation [29,30]. While the ROS induces unsaturated lipid peroxidation, it yields the highly reactive lipid peroxyl radicals, which are quickly neutralized by α-tocopherol through the donation of its hydrogen to the peroxyl radicals, thus holding their propagations within membranes and circulating lipoproteins. The reduced ubiquinol then helps regenerate the α-tocopherol antioxidant capacity through their redox interactions. Thereafter, the ubisemiquinone intermediate can either react with the oxygen molecule and produce the superoxide anion radicals or oxidize further to the fully oxidized ubiquinone that does not react with oxygen. Reduced NADP (NADPH), glutathione, and other antioxidants such as vitamin C then help to regenerate the oxidized ubiquinone and α-tocopherol back and maintain their reduction states [13,31,32,33]. In this integrated manner, CoQ_10_ limits the production of lipid peroxyl radicals and protects the circulating lipoproteins, the cellular membrane proteins, the mitochondrial DNA, and the ETC membranes [26,34,35,36].

For oral CoQ_10_ supplementation, a 2020 meta-analysis of 17 randomized clinical trials (RCT) documented its antioxidant potentials, comprising the reduction of membrane oxidative damage level, the enhancement of total antioxidant capacity, and the activation of antioxidant defense system enzymes [30]. As a dietary supplement, the antioxidant capability of CoQ_10_ might provide benefits to clinical conditions with underlying oxidative stress pathophysiology.

### 3.3. Other Physiological Roles of CoQ_10_

CoQ_10_ also serves as the structural component of the ETC membrane supercomplexes that ascertains the efficient ETC functions and prevents the leakage of the electron from the respiratory chain [28,37,38,39]. The combined result of its structural contribution, lipid peroxidation protection, ROS scavenging, and uncoupling protein activations contribute to the pivotal role of CoQ_10_ in mitochondrial membrane integrity [13,40]. Besides this, the conservation of mitochondrial membrane permeability is also crucial for cellular survival and functions [13,41,42].

Apart from the mitochondria, the containment of highly acidic enzymes within the lipid-membrane boundary of lysosomes requires the CoQ_10_-induced intermembrane proton gradient [8,43]. Furthermore, CoQ_10_–redox interaction maintains the balance of cytosolic redox intermediates such as NADH, NADPH, and FADH_2_. The CoQ_10_-mediated reaction also supplies orotate for the de novo pyrimidine synthesis through the oxidative activity of dihydroorotate dehydrogenase [44]. These intracellular redox balances influence several cellular signalings and gene transcriptions. Such homeostasis modulates apoptosis, bioenergetics, cell growth, and inflammatory responses [28,45].

Interestingly, the oral CoQ_10_ supplementation showed different 115 gene expressions in the muscle tissue samples from aged individuals compared to their placebo controls [46]. These findings supported the diverse physiologic roles of CoQ_10_ and the potential benefit of its intervention. Several clinical studies also reported that oral CoQ_10_ supplementation showed anti-inflammatory effects, including the reduction of tumor necrosis factor-alpha (TNF-α), interleukin-6 (IL-6), and C-reactive protein (CRP) [47,48,49,50]. This immunomodulatory potential of CoQ_10_ supplementation also suggests its potential benefit to various immune-mediated clinical conditions.

## 4. CoQ_10_ Supplementation in Specific Medical Conditions

Cumulative evidence supports the association of low plasma CoQ_10_ levels in several medical conditions such as diabetes mellitus, cancers, and congestive heart failure [2,51,52,53]. Several studies have also explored the role of oral CoQ_10_ supplementation in various conditions. Despite the concerns that oral CoQ_10_ supplementation may excessively raise the tissue CoQ_10_ concentrations in humans [43,54,55], the tissue CoQ_10_ uptake in a healthy individual is relatively low due to its ongoing endogenous biosynthesis [56]. Following the oral supplementation, the plasma CoQ_10_ level appeared to reach the plateau at the dosage of 2400 mg/day while the tissue CoQ_10_ uptakes appeared at a relatively higher plasma concentration than this level [57,58,59]. The tissue uptakes probably increase under the pathological CoQ_10_ deficit [60]. To support this notion, CoQ_10_ supplementation in elders who underwent cardiac interventions showed increased CoQ_10_ uptake in their cardiac tissue samples [61,62]. As for the potential adverse effects of oral CoQ_10_ supplementation, previous studies reported no major side effects after eight months for the dosage of 3000 mg/day, sixteen months for 1200 mg/day, and thirty months for 600 mg/day [58,59,63,64]. Nevertheless, the documented minor gastrointestinal symptoms included nausea, diarrhea, low appetite, heartburn, and discomfort, notably when the daily dosage exceeded 200 mg/day. The two or three daily divided doses minimized most of these side effects [65]. The concurrent intake of high-fat meals also facilitates CoQ_10_ absorption and reduces gut-related symptoms.

Many clinical studies explored the role of CoQ_10_ supplementation, either as a single intervention or as a combination with other nutrients, in several medical conditions. In general, the outcomes of single CoQ_10_ supplementation from these trials were largely inconsistent. We herein explored previous CoQ_10_ supplementation studies and deduced the potential contributing factors to the interventional outcomes.

### 4.1. Single CoQ_10_ Supplementation

#### 4.1.1. Single CoQ_10_ Supplementation in the Primary CoQ_10_ Deficiencies

The primary CoQ_10_ deficiencies are genetic conditions with mutations in one of the nine CoQ_10_ biosynthetic genes [5]. These mutations lead to the disruption in the mitochondria respiratory chain functions with the clinical phenotypes of multisystem involvement [66,67]. Despite the incurability of the primary CoQ_10_ deficiencies, studies reported a partial improvement of muscular and neurological symptoms in some patients with oral CoQ_10_ supplementation [68]. A systematic review of the intervention in patients with primary CoQ_10_ deficiencies, a total of 89 cases, reported symptom improvements in 27% of patients. Five cases even deteriorated after discontinuing CoQ_10_ supplementation [69]. The intervention on this genetic condition generally required a high dosage, ranging from 5 to 50 mg/kg/day, of oral CoQ_10_ supplementation to achieve favorable responses [70].

#### 4.1.2. Single CoQ_10_ Supplementation in Healthy Adults and Athletes

A single bout of vigorous exercise in young athletes induces a rapid decrease in plasma CoQ_10_ level and one month of supplementation minimized that effect [16]. Two weeks of CoQ_10_ supplementation, 200 mg/day, before performing strenuous exercise sessions showed antioxidant benefits in a group of 100 healthy and trained adults in a 2016 RCT. These oxidative stress protections included the reduction of oxidative damage markers and enhanced antioxidant enzyme activities [71]. A systematic review of 13 clinical studies also supported these findings [72]. A recent 2022 RCT also supported the improved endothelial reactivity from CoQ_10_ supplementation in 20 healthy adults [73]. Apart from the antioxidant protections, a trial on oral supplementation during the periods of high-intensity exercises also showed benefits in the modulation of inflammatory signaling, the pro- and anti-inflammatory cytokines released, together with a potential pro-angiogenic effect on hematologic parameters such as hemoglobin, red blood cell number, vascular endothelial growth factor (VEGF), and epidermal growth factor (EGF) [74].

On the contrary, a cross-over, double-blind, and placebo-controlled trial did not show significant changes in the aerobic capacity and lipid peroxidation markers in 19 trained adults, 11 young and 8 older, after six weeks of ubiquinone supplementation at 120 mg/day [75]. Despite the recovery of exercise-induced depleted plasma CoQ_10_ level, the athletes’ biomarkers of muscular damage and physical performance remained unchanged [16]. Several RCTs also failed to support the benefits to the anaerobic performance during high-intensity training from oral supplementation [76,77,78]. During the intensive Kendo training, CoQ_10_ supplementation did not ameliorate exercise-induced muscle damage in a study of a four-day training period but showed a protective effect in another study of a six-day training period [79,80]. The high-altitude trekkers did not obtain a protective effect for cardiac alterations after their 17-day trek to Everest Base Camp [81]. An 8-week supplementation in ten trained cyclists did not show measurable effects on their performance, VO_2_max, or lipid peroxidation [82]. Another study on endurance athletes also showed no significant changes in their cardiorespiratory fitness parameters and blood metabolic markers [83].

Nonetheless, 25 Finnish top-level cross-country skiers achieved significant improvement in all physical performance indices with supplementation [84]. Short-term CoQ_10_ supplementation in elite swimmers modulated their energy metabolism, enhanced antioxidant capacity, and prevented the elevation of lipid peroxidation and cardiac damage markers [85,86,87,88]. Another study on a six-day Kendo training period reported the downregulation of toll-like receptor 4 (TLR-4) in monocytes in the athletes who took the supplementation for 20 days [89].

Several RCTs on oral CoQ_10_ supplementation in healthy adults and athletes yielded inconsistent outcomes for their exercise performance, muscle damage prevention, antioxidant protection, and immunologic modulations. Up to now, only one systematic review of supplementation in healthy adults has been published, with no relevant meta-analysis. The outcome discrepancies might depend on contributing factors such as individual host metabolic status, type and intensity of exercises, timing and dosing of supplementation, or other interacting nutrients.

#### 4.1.3. Single CoQ_10_ Supplementation in Elders

Human CoQ_10_ biosynthesis decreases with advancing age [17,34]. At the age of 80, the myocardial CoQ_10_ production is only half compared to levels in a 20-year-old person [8,17]. The elderly also extensively use many prescribed medications, including statin, bisphosphonates, and β-blockers, which interfere with endogenous CoQ_10_ biosynthesis [20,21,90,91,92,93]. In addition to their eating pattern alteration and presumably compromised metabolic status, the likelihood of low blood levels of CoQ_10_ in elders is not uncommon [5].

In addition to the Mediterranean diet, healthy elders with oral CoQ_10_ supplementation showed significant benefits in their redox-state parameters, postprandial metabolism of advanced glycation end products (AGEs), metabolomic profiles, and the modulation of gene expressions that involved anti-inflammatory, endoplasmic reticulum stress, DNA repair, and antioxidant functions [94,95,96,97,98,99].

Even though there was no available meta-analysis, we found no study not supporting the benefits of oral CoQ_10_ supplementation in elders as compared to the trials in healthy adults and athletes. These different outcomes could partly be due to the temporary nature of depleted CoQ_10_ levels in healthy adults and athletes after their physical exertions, while the depleted CoQ_10_ levels in elders are a result of their ages, illnesses, and metabolic statuses.

#### 4.1.4. Single CoQ_10_ Supplementation in Metabolic and Immune-Related Disorders

The early two systematic reviews in 2003 and 2009 failed to conclude whether the oral CoQ_10_ supplementation had any effect on blood pressure [100,101]. Contrarily, the following four meta-analysis studies, in 2012, 2016, and two in 2018, of 5, 14, 17, and 21 RCTs, respectively, reported that CoQ_10_ supplementation improved endothelial function, reduced systolic blood pressure, fasting blood glucose, and serum triglycerides, and improved lipid profiles [102,103,104,105]. While the 2015 RCT suggested that daily supplementation could help decrease the pro-inflammatory cytokines [106], a 2011 RCT in 51 obese subjects did not find an association between the supplementation and lipid profile, oxidative and inflammatory markers, arterial stiffness, and fatigue indices [107].

Contrarily, another 2019 meta-analysis of 17 RCTs did not support the benefits of CoQ_10_ supplementation on the body weight and BMI of patients [108]. In dyslipidemic individuals, the 2016 and 2018 RCTs on CoQ_10_ supplementation showed benefits in the improvements of lipid and glycemic profiles, antioxidant capacity, endothelial reactivity, and blood pressure [109,110]. A 2000 RCT in 12 hypercholesterolemic young adults did not show a significant effect on endothelial dysfunction [111]. Conflictingly, another 2020 RCT in 51 dyslipidemic subjects had benefits of endothelial dysfunction amelioration from CoQ_10_ supplementation [112]. We did not find a meta-analysis on the supplementation impacts on dyslipidemic subjects.

In diabetes patients, the clinical impacts of CoQ_10_ supplementation were also inconsistent despite the significant association of low CoQ_10_ levels in these patients [113,114,115]. A 2015 meta-analysis of 7 RCTs concluded no benefit on glycemic and lipid profiles in diabetes subjects [19]. Nevertheless, three RCTs, one in 2017 and two in 2018, on overweight or obese diabetic patients showed reduced glycosylated hemoglobin levels, reduced insulin levels, and increased antioxidant enzyme activities [116,117,118]. A 2018 meta-analysis of 13 RCTs suggested the benefits of CoQ_10_ supplementation on glycemic and lipid profiles in type 2 diabetic patients [119]. The single CoQ_10_ supplementation, 400 mg, also improved the visual acuity, intraocular pressure, and oxidative stress biomarkers in a 2016 RCT of patients with diabetic retinopathy [120]. A cell line study also demonstrated the CoQ_10_ protective effects on retinal ganglion cells from intraocular-pressure-induced hypoxia and subsequent oxidative stress, which are part of glaucoma pathogenesis [121,122].

In patients with coronary artery disease, a 2018 meta-analysis of eight RCTs reported the effects of supplementation on lowering total cholesterol and increasing high-density lipoprotein-cholesterol levels, but no changes in low-density lipoprotein-cholesterol and lipoprotein(a) levels [123]. Another 2019 meta-analysis of 13 RCTs documented the increased antioxidant enzyme activities and decreased oxidative damage markers despite the nonsignificant changes in pro-inflammatory cytokines and CRP [124].

The benefits of oral CoQ_10_ supplementation were likely evident in advancing clinical stages such as congestive heart failure. A 1997 meta-analysis of 14 RCTs concluded the benefits of improved hemodynamic cardiac parameters such as stroke volume, cardiac output, ejection fraction, cardiac index, and end-diastolic volume index [125]. While a 2014 pooled analysis of seven RCTs concluded neither benefits nor harms of the supplementation in patients with heart failure [126], the 1993 and 2020 RCTs supported the supplementation’s benefit on improved endothelial function, reduced hospitalization, and reduced serious complications in patients with heart failure [127,128].

The CoQ_10_ supplementation in patients with chronic kidney disease could improve some of their metabolic profiles, such as creatinine, lipid parameters, and oxidative damage markers, as reported in a 2018 meta-analysis of seven RCTs [129]. In diabetic nephropathy, CoQ_10_ supplementation modulated gene expression of peroxisome proliferator-activated receptor-γ, interleukin-1, and TNF-α, together with the favorable impacts on glucose metabolism [130,131]. The supplementation in diabetic hemodialysis patients also provided benefits to insulin metabolism, with increased antioxidant capacity and decreased CRP, although there were no changes in exercise performance, diastolic heart function, fasting glucose, glycosylated hemoglobin, lipid profile, and oxidative damage markers [132,133,134,135].

For nonalcoholic fatty liver disease patients, CoQ_10_ supplementation also provided benefits in several anthropometric and biochemical parameters, including waist circumference, liver aminotransferases, CRP, TNF-α, adiponectin, leptin, vaspin, chemerin, and pentraxin 3 [136,137].

In chronic inflammatory conditions, the 2019 meta-analysis of nine RCTs supported the significant impacts of CoQ_10_ supplementation on the modulation of pro-inflammatory signals, including TNF-α and IL-6 [50]. Several RCTs on other immune-related conditions, including fibromyalgia, rheumatoid arthritis, and multiple sclerosis, supported the immunomodulatory effects of supplementation [138,139,140,141,142]. For instance, a 2015 RCT of 500 mg CoQ_10_ supplementation documented the amelioration of pro-inflammatory biomarkers such as TNF-α, IL-6, and MMP-9 in patients with relapsing-remitting multiple sclerosis [143].

Despite the inconsistent results on single CoQ_10_ supplementation clinical trials in various metabolic disorders, the following meta-analysis tended to show more positive metabolic benefits in patients with advanced clinical stages, such as cardiovascular diseases, heart failure, or kidney failure, than the ones in early clinical phases, such as hypertension or dyslipidemia.

#### 4.1.5. Single CoQ_10_ Supplementation in Those Who Take Prescribed Medications

CoQ_10_ biosynthesis requires an enzyme in the mevalonate pathway, 3-hydroxy-3-methylglutaryl (HMG)-CoA reductase, which is the common enzyme for cholesterol biosynthesis [144,145]. Statin is a commonly prescribed lipid-lowering medication that inhibits HMG-CoA reductase. Statin is used in combination with other prescribed medications to treat various conditions that particularly co-exist in aging adults, therefore contributing to the decreased plasma CoQ_10_ levels [20,21]. Nitrogen-bisphosphonates (N-BPs) is another prescribed medication in elders, commonly used for the treatment of age-related osteoporosis [92]. N-BPs inhibit farnesyl pyrophosphate synthase, another enzyme in the CoQ_10_ biosynthesis, therefore affecting the circulating CoQ_10_ level as well [146]. Moreover, women with osteoporosis who were treated with N-BPs showed a concurrent reduction of the γ-tocopherol level, a crucial nutrient in integrated antioxidant defenses [92]. These combined effects of prescribed medications could potentiate the adverse consequences of depleted CoQ_10_ levels.

Despite the established correlation of statin-induced myopathy, a 2015 meta-analysis of six RCTs did not support the post-interventional benefits of CoQ_10_ supplementation [147]. Contrarily, another 2018 meta-analysis of 12 RCTs supported the amelioration of statin-associated myopathy [148]. Derosa et al. also reported the significant mitigation of statin-related side effects with liquid CoQ_10_ supplementation for three months in the 2019 RCT of 60 Caucasian patients [149]. On the contrary, a recent 2022 retrospective multicenter study did not find any benefits of the supplementation to statin-associated muscle symptoms [150].

Despite the likelihood of low CoQ_10_ levels in subjects who take medications, the benefits from the supplementation studies were also inconsistent, even though the available meta-analysis seemed to support the intervention. Other contributing factors, such as the concurrent depletion of multiple interacting nutrients within the integrated antioxidant network, might hinder the outcome of single CoQ_10_ supplementations in these subjects. Unfortunately, these potential confounders were not controlled in the participants of previous clinical trials.

#### 4.1.6. Single CoQ_10_ Supplementation in Neurological Disorders

Neurogenerative disorders such as Parkinson’s disease (PD) and Alzheimer’s disease (AD) share some pathophysiologies, including mitochondrial dysfunction and oxidative stress [151]. Increased oxidative stress was shown as the significant elevation of the serum oxidized CoQ_10_ levels in patients with amyotrophic lateral sclerosis, compared to their age-matched healthy controls [152]. Nevertheless, many RCTs on these neurodegenerative subjects showed conflicting results similar to other single CoQ_10_ supplementation trials. Despite the assurance of its safety and tolerance in these conditions, a 2017 meta-analysis failed to suggest the intervention’s benefits [153].

In AD patients, early 1994 and 1998 RCTs demonstrated the clinical benefits of single CoQ_10_ supplementations for memory, attention, orientation, and disease progression [154,155]. However, the 2003 RCT did not find significant differences between the study groups [156]. A dose of 360 mg CoQ_10_ for 4 weeks in patients with PD provided moderate benefits on scored PD symptoms and visual function [157]. In neuromuscular disorders such as Huntington’s disease (HD), a 2017 large multicenter RCT on high doses of CoQ_10_, at 2400 mg a day, did not significantly slow the progressive functional declination in these patients compared to their controls [158,159].

For neurological conditions, the effectiveness of CoQ_10_ supplementation was rather promising in small-scale clinical studies. However, larger-scale RCTs failed to provide consistent effects. The advanced nature of these neurological conditions, with established neuronal losses at the time of diagnosis, could partly contribute to these disparities in the results of a single CoQ_10_ intervention. Unlike the previously mentioned trends in metabolic diseases, a nutrient intervention is less likely to be effective in advanced neurological conditions.

#### 4.1.7. Single CoQ_10_ Supplementation in Cancers

Low circulating CoQ_10_ levels are associated with increased breast cancer risk [160]. In vivo CoQ_10_ supplementation appeared to enhance the DNA repair enzyme activities and protect the DNA from oxidative damage [22]. A CoQ_10_ intervention, at 300 mg/day for 12 weeks, significantly improved the antioxidant capacity and reduced oxidative damage and inflammatory levels in post-surgical patients with hepatocellular carcinoma [161]. However, a 24-week-supplementation RCT did not show improvements in fatigue and other quality of life parameters in women with breast cancer [162]. A 2004 systematic review of six studies, could not conclude whether CoQ_10_ supplementation could improve the tolerability of cancer treatments [163]. The benefits of single CoQ_10_ supplementation in cancers are either preventive or protective rather than curative.

### 4.2. Combined CoQ10 Supplementation with Other Nutrients

Human metabolism fundamentally requires support from an integrated nutrient network. Abided by this fact, CoQ_10_ contributes its essential role by coordinating with other macro- and micronutrients in the bioenergetic and antioxidant circuits [5]. Genetic predisposing conditions are the only exception to this integrated function, where the prone individuals are subjected to a specific nutrient inadequacy, which may require high-dose single nutrient intervention to alleviate the situation, as previously mentioned in primary CoQ_10_ deficiencies. Patients with chronic illnesses largely endure concurrent multiple nutrient insufficiencies [164]. Hence, it is understandable why single CoQ_10_ supplementation yielded inconsistent outcomes, particularly in hosts with severely compromised nutrient reserves. For this reason, combining CoQ_10_ supplementations with other nutrients could potentially augment the clinical benefits in these situations [6,165,166,167].

Accordingly, studies in rat models and cell lineages reaffirmed that the combination of CoQ_10_, multivitamins, and minerals protected organ damage through the reduction of oxidative damage and inflammation [168,169,170,171]. Several human trials also reported oral combined supplementation of CoQ_10_ and other nutrients with beneficial responses [166,172,173,174]. Among the previous trials, the familiar combined supplementation was CoQ_10_ and selenium, an important cofactor of glutathione peroxidase—a key antioxidant enzyme. However, numerous studies use different nutrient combinations, which generally comprised those that supported mitochondrial bioenergetic and antioxidant networks, including vitamin Bs, vitamin C, vitamin E, selenium, zinc, lipoic acid, L-carnitine, and taurine [175].

#### 4.2.1. The Combined Supplementation of CoQ_10_ in Healthy Adults and Athletes

In a 2016 RCT of healthy volunteers, the 6-month combined supplementation of CoQ_10_, multivitamins, and minerals reduced nitrosative stress and improved mitochondrial bioenergetics [176]. A total of 83 infertile males taking a combined supplementation of CoQ_10_, l-carnitine/acetyl-l-carnitine, l-arginine, glutathione, zinc, vitamin B9, vitamin B12, and selenium improved their sperm quality and increased the pregnancy rate in a 2020 RCT [177]. Two meta-analyses, in 2018 and 2019, of 15 and 18 RCTs, respectively, suggested the favorable effects on sperm quality parameters of infertile males from CoQ_10_ and other nutrients such as selenium, zinc, l-carnitine, and omega-3 fatty acids [178,179].

However, triathletes with the combined supplementation of CoQ_10_, vitamin C, and alpha-tocopherol did not gain benefits in their exercise performance [180]. In a 2005 RCT, the combined supplementation of CoQ_10_, alpha-lipoic acid, N-acetyl cysteine, vitamin C, alpha-tocopherol, manganese, and selenium did not protect against exercise-induced DNA damage [181]. Prior supplementation of combined CoQ_10_ and alpha-tocopherol also did not attenuate either lipoprotein oxidation or muscle damage during exhaustive exercise in marathon runners [182]. Contrarily, a mixture of CoQ_10_, multivitamins, and minerals helped lower the oxidative damage markers following a 60-min soccer match after 3-month supplementation in pre-professional footballers [183]. The cocktail of CoQ_10_, vitamin C, and alpha-tocopherol also raised the LDL antioxidant potential in endurance athletes [184].

The combined supplementation likely improved the favorable outcomes in healthy adults, while the outcomes from clinical trials in athletes were still inconsistent. The differences in types and intensities of exercise could partly account for these discrepancies. However, a systematic review or meta-analysis on athletic intervention is not yet available.

#### 4.2.2. The Combined Supplementation of CoQ_10_ in Elders

The 6-month oral supplementation of combined CoQ_10_, multivitamins, and selenium significantly elevated the blood CoQ_10_ level in healthy elderly women [185]. A 2015 RCT on active 48-month supplementation of CoQ_10_ and selenium in Swedish elders showed the reduction of CRP and P-selectin levels, together with the increased levels of insulin-like growth factor 1 and insulin-like growth factor binding protein 1 [165,186]. The metabolomic profiles of these elders suggested changes in the pentose phosphate, the mevalonate, the beta-oxidation, and the xanthine oxidase pathways, together with the changes in the urea cycle and the increased neurotransmitter precursors after 18 months of intervention [187]. Elders in the supplementation group also had an increased number of days out of the hospital and a slowed deterioration of health-related quality of life scores [188]. The 12-year follow-up of these 443 elders, who continued the combined supplementation for four years, still had significantly reduced cardiovascular mortality [174]. Apart from the combination with selenium, the 12-week mixed supplementation of CoQ_10_, essential amino acids, creatine, and vitamin D also showed positive effects on the muscle mass, strength, power, and visceral adipose tissue of 38 healthy elders in a 2019 RCT [189]. While CoQ_10_ decreased by 40% in elders, the combined supplementation of CoQ_10,_ acetyl-L-carnitine, and omega-3 fatty acids in 106 patients with early age-related macular degeneration improved visual functions and stabilized fundus alterations in an RCT [190,191].

Although without available meta-analysis, the results from these RCTs on elders provided consistent trends of benefits from combined supplementation of CoQ_10_.

#### 4.2.3. The Combined Supplementation of CoQ_10_ in Metabolic and Immune-Related Disorders

A 2016 meta-analysis of 14 RCTs of the formulated supplementation of CoQ_10_, red yeast rice, berberine, policosanol, astaxanthin, and folic acid suggested its benefits on lipid and glucose profiles [192]. Two RCTs, in 2017 and 2019, in patients with dyslipidemia and pre-hypertension. respectively, also documented the positive impacts on lipid and glucose profiles, CRP, and liver transaminase with the combination of CoQ_10_, red yeast rice, and other nutrients [193,194]. For patients with metabolic syndrome, the combination of CoQ_10_ and red yeast rice provided benefits to their blood pressure, lipid, and glycemic biomarkers in another 2018 RCT [195]. A recent 2021 meta-analysis of 12 RCTs also supported the beneficial impacts on serum lipids, glucose, and CRP with the combination of CoQ_10_, red yeast extract, policosanols, berberine, folic acid, and astaxanthin [196].

In patients with nonproliferative diabetic retinopathy, their plasma CoQ_10_ levels were decreased as compared with the healthy controls [18]. The supplementation of CoQ_10_, pycnogenol, and vitamin E led to decreased circulating free oxygen radical levels, although there was no significant change in central macular thickness at six months, compared to the controls [197]. The local application of visudrop, the combination of CoQ_10_ and vitamin E, during cataract surgery significantly reduced postoperative corneal edema and pain, with enhanced vision outcomes [198]. The application of an ophthalmic solution containing CoQ_10_ and vitamin E in patients with open-angle glaucoma showed benefits on the inner retinal function, with subsequent enhanced visual cortical responses [199].

For patients with cardiovascular disease, a 2006 narrative review suggested CoQ_10_ as one of the first-line conditionally essential nutrients, along with l-arginine, l-carnitine, and propionyl-l-carnitine, while the supplementation of these nutrients could provide favorable clinical impacts [200]. A pilot study on combined supplementation of CoQ_10_, magnesium, potassium, vitamin B12, folic acid, and niacin reported improved left ventricular diastolic function parameters and fasting insulin levels in patients with cardiac arrhythmia [201]. The elders with chronic heart failure improved their left ventricular functions and quality-of-life parameters with the combined supplementation of CoQ_10_, multivitamins, and minerals [202]. Two RCTs, in 2007 and 2011, in chronic heart failure patients, also supported the favorable effects on their physical performance parameters and inflammatory signal modulation, from the combined supplementations of either CoQ_10_ and creatine or CoQ_10_ and l-carnitine [203,204].

The supplementation of CoQ_10_, together with multivitamins and minerals modulated the biomarkers of immunologic and autonomic dysfunctions in patients with end-stage renal disease [205]. Two months with the combined CoQ_10_ and creatine supplementation also helped to improve functional performance, body composition, and dyspnea symptoms in patients with the chronic obstructive pulmonary disease [206]. On the contrary, the combination of CoQ_10_ and omega-3 fatty acids did not provide a significant change in plasma myeloperoxidase level, a mediator of chronic inflammation, in patients with chronic kidney disease, in a 2018 RCT [207].

For immune-related disorders, psoriatic patients showed increased activities of antioxidant defenses in the circulating granulocytes and the affected epidermis with the combined supplementation of CoQ_10_, vitamin E, and selenium [208]. Patients with chronic fatigue syndrome improved their bioenergetic biomarkers and age-predicted maximum heart rate during a cycle ergometer test with CoQ_10_ and NADH supplementation [209,210]. A total of 130 adults with migraine also significantly reduced the pain intensity with the supplementation with CoQ_10_, riboflavin, and magnesium in an RCT from Gaul et al. [211]. The benefits of migraine prophylaxis were also supported either with CoQ_10_ and l-carnitine or CoQ_10_ and curcumin interventions in 2019 and 2021 RCTs [212,213].

Even though a recent meta-analysis was still not available, the trends of previous studies were encouraging for clinical benefits in various metabolic and immune-related disorders with combined CoQ_10_ supplementation.

#### 4.2.4. The Combined Supplementation of CoQ_10_ in Those Who Take Prescribed Medications

The combined CoQ_10_ and selenium supplementation substantially elevated the relevant serum levels in patients taking statins but did not significantly mitigate their myopathy symptoms in two 2013 RCTs [214,215]. However, three months on CoQ_10_ and carnitine supplementation showed a significant reduction of serum lipoprotein(a) in hemodialysis patients with statin therapy [216]. With the context of a limited study number, the conclusion for the impacts of combined supplementation on these patients warrants future trials.

#### 4.2.5. The Combined Supplementation of CoQ_10_ in Neurological Disorders

There were also few studies on mixed CoQ_10_ intervention in various neurological conditions. According to the Alzheimer’s Disease Cooperative Study, the combined CoQ_10_ and vitamin E, vitamin C, and α-lipoic acid did not influence the levels of amyloid or tau proteins in cerebrospinal fluid. Interestingly, the intervention group had a more rapid cognitive declination than their controls, which raised the safety concerns of this mixed supplementation [217]. Thus far, the impacts of combined CoQ_10_ supplementation were still inconclusive in these complex clinical conditions.

#### 4.2.6. The Combined Supplementation of CoQ_10_ in Cancers

Patients with end-stage cancers significantly increased their life expectancy, from an average of 12 to 17 months, with a combined supplementation of CoQ_10_ and antioxidant mixture [218]. In breast cancer patients under tamoxifen treatment, the daily supplementation of CoQ_10_, riboflavin, and niacin decreased their pro-inflammatory cytokine levels, increased the DNA repair enzyme levels, and suppressed the DNA methylation pattern, which might lead to tumor burden reduction [219,220]. In a multicenter RCT, 57 women with breast cancer women, who took combined supplementation of CoQ_10_ and L-carnitine, reported relieved cancer-associated fatigue symptoms [162,221]. However, several RCTs in high-risk people or patients with prostate cancers did not support the benefit of combined supplementation of CoQ_10_, vitamin E, selenium, and vitamin C, along with several phytochemicals [222,223].

## 5. Discussion

The inconsistent results of CoQ_10_ interventions implied the presence of unaccounted factors that contributed to clinical outcomes. After reviewing the participants’ status in previous CoQ_10_ clinical trials, we herein proposed two potentially confounding aspects, i.e., differences in host metabolic status and the need for CoQ_10_ interacting nutrients.

Human metabolism fundamentally relies on host macro- and micronutrient reserves. Depleted host nutrient reserve leads to metabolic triage of nutrients toward the preservation of short-term metabolic survival, usually at the cost of compromised long-term health [224,225]. The protein deformations, with altered enzyme binding constants for various coenzymes, underly these nutrient triage processes [226]. Compromised host nutrients induce metabolic triage and accelerate the pathophysiologies of degenerative and metabolic diseases [227]. Therefore, nutrient interventions could hinder mitochondrial decay and delay age-associated illnesses [225].

Even the so-called healthy subjects were still prone to conditional micronutrient inadequacies following intense physical activities, despite their good metabolic statuses and no established clinical diagnosis at baseline. The combined nutrient interventions hold better chances to address the conditional nutritional insufficiencies than a single nutrient. To support this notion, previous studies showed the favorable trends of combined CoQ_10_ intervention in healthy adults and athletes performing exercise sessions, as compared to single supplementation.

Elders and patients with diagnosed metabolic and immune-related disorders likely had compromised metabolic status, along with multiple nutrient insufficiency. The depleted nutrient reserves increased with the advancement of these chronic situations. Hosts with specific nutrient depletion, such as primary CoQ_10_ deficiencies, benefited from single CoQ_10_ supplementation, even though the outcomes were mostly palliative, not curative. The single intervention was also beneficial in hosts with early stages of declined metabolic status, such as the elderly. The benefits decreased with the advancement of metabolic conditions, as seen in patients with diabetes, cardiovascular diseases, or kidney failure. Despite the improvement of some surrogate biomarkers such as proinflammatory cytokines, antioxidative capacities, and lipid or glycemic profiles, the positive trends in clinical outcomes were less promising with single supplementation. Contrarily, combined CoQ_10_ interventions provided more encouraging results in hosts with impaired metabolic status due to the readily available interacting nutrients in the formulations.

However, both CoQ_10_ interventions would be less beneficial in far-advanced conditions with established damage such as neurodegenerative conditions or cancers. The results of both single and combined supplementation, at best, affected some surrogate biomarkers but not the overall clinical outcomes. Therefore, nutrient interventions are preventive or protective rather than curative measures.

## 6. Conclusions and Future Perspectives

CoQ_10_ is a compound with crucial roles in mitochondrial bioenergetics, membrane antioxidant protection, and many cellular signaling regulations. However, no single nutrient could magically drive whole physiological processes. Single CoQ_10_ supplementation will be beneficial only for hosts that specifically require it, such as hereditary CoQ_10_ deficiencies. The single intervention will be less promising when the host metabolic status worsens with the likelihood of multiple nutrient insufficiencies. On the contrary, the mixed CoQ_10_ supplementation with other interacting nutrients will create more promising impacts in hosts with compromised nutrient reserves. However, the results of either single or combined intervention will be less promising in far-advanced conditions with established damage.

With the limited amount of high-level evidence, such as provided by systematic reviews and meta-analyses, we could only conclude that the considerations of whether to take supplementation varied by the individuals’ metabolic status and their nutrient reserves, which span across the continuum of metabolic triage processes that lead to chronic health issues. Future studies are warranted, particularly for the RCT with the design to control the host metabolic and nutrient status of participants and the meta-analysis of upcoming CoQ_10_ studies on each subject’s metabolic status.

## Data Availability

Not applicable.
